# Thalamocortical connectivity associated with fatigue after accounting for depressive symptoms in stroke survivors and controls

**DOI:** 10.1016/j.nicl.2026.104063

**Published:** 2026-10-08

**Authors:** Joan Stilling, Christie Gillies, Ji Hyun Kim, Keith Jamison, Amy Kuceyeski

**Affiliations:** a Weill Cornell Medicine, Department of Rehabilitation Medicine, New York, NY, United States of America; b Weill Cornell Medicine, Department of Neuroscience, New York, NY, United States of America; c New York Medical College, Valhalla, NY, United States of America; d Weill Cornell Medicine, Department of Radiology, New York, NY, United States of America; e Cornell University, Computational Biology & Statistics and Data Science, Ithaca, NY, United States of America

**Keywords:** Stroke, Fatigue, Functional connectivity, Brain mapping, Depression, Thalamus, Recovery of function

## Abstract

**Background:**

Post-stroke fatigue is common and disabling, yet rarely distinguished from co-occurring depression, limiting understanding of its neural substrates. We examined resting-state fMRI correlates of post-stroke fatigue while accounting for depressive symptoms.

**Methods:**

We studied 103 stroke patients and 29 age-matched controls from a longitudinal neuroimaging cohort. Fatigue was assessed via the SF-36 Vitality subscale and depression via the Geriatric Depression Scale - Short (GDS-15). Linear mixed-effects models evaluated associations between vitality and resting-state functional connectivity, adjusting for depression and covariates. Residualized vitality models served as sensitivity analyses to isolate fatigue variance independent of depressive symptoms, with FDR correction applied within feature families.

**Results:**

In depression-adjusted models, higher vitality (lower fatigue) was associated with increased frontoparietal-limbic connectivity (β = 0.0013, FDR q = 0.048), though this did not survive depression residualization. In a residualized sensitivity analysis, higher vitality (lower fatigue) was associated with reduced thalamus-insula and thalamus-ventral attention network connectivity (thalamus-insula: β = −0.0071, FDR q = 0.011; thalamus-VAN: β = −0.0043, FDR q = 0.012); these associations were attenuated and did not survive correction in the primary depression-adjusted model (q ≈ 0.075).

**Conclusions:**

Fatigue was associated with both frontoparietal-limbic and thalamocortical connectivity, although the thalamic associations were attenuated after adjustment for depressive symptoms in the primary analysis and were most evident in residualized sensitivity analyses. These findings suggest that fatigue and depressive symptoms may involve both shared and potentially distinct neural correlates. Point estimates were smaller in stroke participants than controls, providing no evidence that the observed associations were stroke-specific. Future work should determine whether these relationships are unique to stroke or reflect broader neural correlates of fatigue across clinical populations.

## Highlights

• Fatigue is common after stroke, but its neurobiological basis remains poorly understood.
• Frontoparietal-limbic connectivity was associated with shared fatigue-depression variance across groups.
• Greater thalamus-insula and thalamus-attention connectivity was associated with greater fatigue in sensitivity analyses.
• Thalamic fatigue associations did not significantly differ by stroke status, lesion location, or lesion volume.
• These findings support further investigation of thalamocortical circuitry as a correlate of fatigue across populations.

## Graphical abstract

**Figure:**
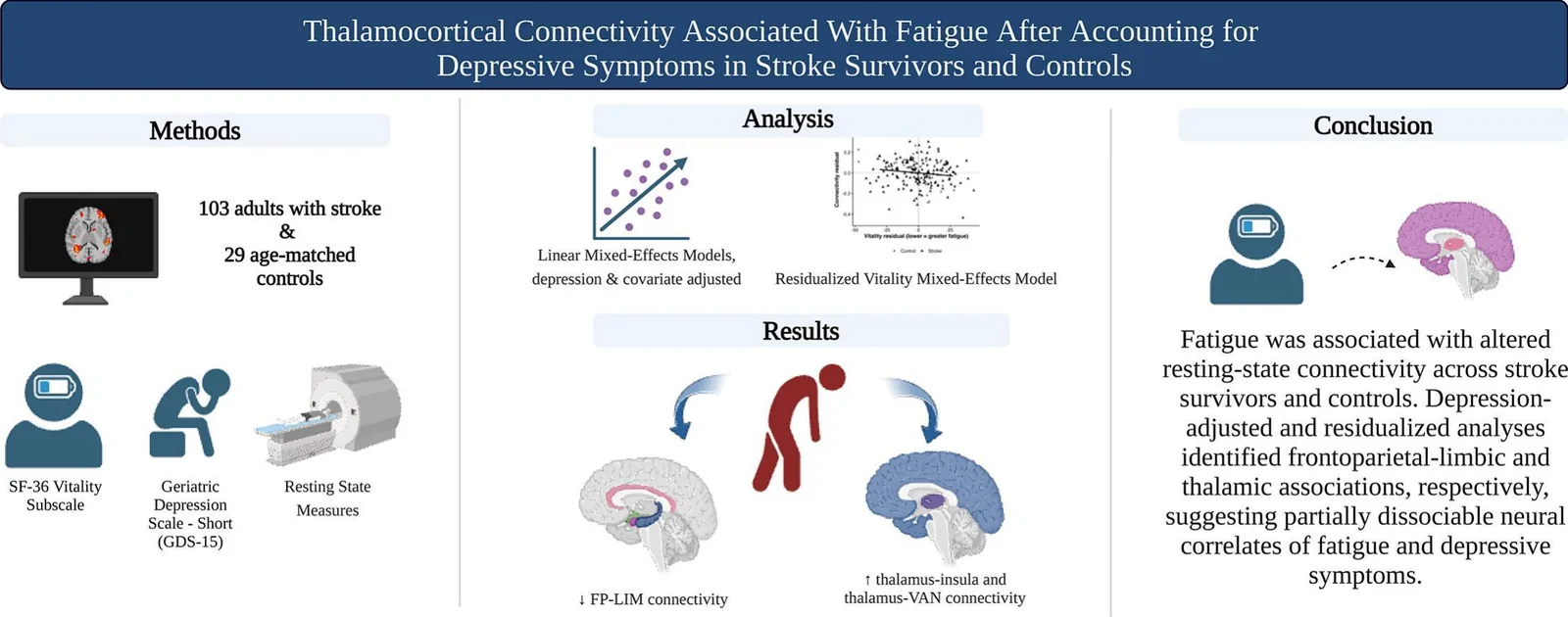

## 1 Introduction

Post-stroke fatigue (PSF) is a common and persistent complication of stroke, affecting an estimated 42–53% of survivors (Christensen et al., 2008; Cumming et al., 2016; Paciaroni and Acciarresi, 2019). This range reflects cross-sectional prevalence estimates rather than incidence of new-onset fatigue following stroke, and reported rates vary further with the specific fatigue measure, threshold, and post-stroke timepoint used across studies. PSF is defined as a persistent lack of physical or mental energy that is not relieved by rest and that interferes with daily activities (Paciaroni and Acciarresi, 2019). It is independently associated with reduced functional recovery, lower rates of return to work, and increased mortality (Christensen et al., 2008; Cumming et al., 2016). Despite its prevalence and impact on quality of life, the neural substrates of PSF remain poorly understood, and there are currently limited pharmacological or non-pharmacological interventions with sufficient evidence for its treatment (Bivard et al., 2017; Jolly et al., 2023; Wu et al., 2015a). To date, there is no established gold-standard therapy and management remains multimodal, including education/self-management (i.e., pacing), graded exercise, treating contributing conditions – primarily mood and sleep disorders, and selective use of oral medications that promote wakefulness, such as modafinil (Bivard et al., 2017). Lesion location and size have not been consistently associated with fatigue severity across studies (Boot et al., 2022; Jolly et al., 2023), and serotonin reuptake inhibitors have not shown efficacy for PSF in randomized controlled trials (Wu et al., 2015a), suggesting that PSF may be neurobiologically distinct from post-stroke depression (PSD). However, PSF and PSD frequently co-occur and share overlapping symptom features (i.e., reduced energy, psychomotor slowing), which has complicated both clinical assessment and neuroimaging research (Hackett and Pickles, 2014; MacIntosh et al., 2017; Mutai et al., 2017). Whether PSF has neural correlates that are separable from those associated with depressive symptom burden remains an open question with important implications for treatment development. Both possibilities are clinically informative. If fatigue has partially distinct neural correlates, identifying these circuits could inform interventions targeted to patients whose fatigue occurs without significant depressive symptoms. Conversely, if the underlying mechanisms are substantially shared, such transdiagnostic overlap would itself be informative and could support therapeutic approaches targeting common mechanisms in patients with comorbid fatigue and depression.

Neuroimaging studies of PSF have largely relied on structural MRI to examine lesion characteristics, with inconsistent findings across studies (Boot et al., 2022; Jolly et al., 2023). These approaches are limited in their ability to capture distributed network-level consequences of focal injury (i.e., remote connectivity disruption, diaschisis), which may be more relevant to neuropsychiatric outcomes after stroke. Resting-state functional MRI (rs-fMRI) offers a complementary approach by characterizing large-scale brain network organization without requiring task performance. Prior rs-fMRI studies have noted alterations in default mode and salience network connectivity in participants with PSF, with specific involvement of the precuneus and insula (Cotter et al., 2022; Ondobaka et al., 2022; Ren et al., 2024), but most have not simultaneously modeled depression or used designs capable of separating fatigue-specific neural effects from those shared with depressive burden. This gap limits our ability to identify neural targets that are specific to PSF, hindering the development of effective treatments.

To address this gap, we used a hypothesis-driven approach targeting regions implicated in distributed neural systems supporting arousal, interoception, cognitive control, and effort regulation; processes increasingly linked to pathological fatigue (Kuppuswamy, 2022). We used a multi-staged analytical approach in both stroke survivors and age-matched healthy controls to examine whether associations between fatigue and resting-state functional connectivity persisted after accounting for depressive symptom burden, without assuming that the two constructs are fully separable. We also examined regional signal amplitude (i.e., ALFF and fALFF) and whole-brain node strength to characterize broader patterns of neural disruption and tested whether fatigue-related connectivity was moderated by lesion site or volume in stroke.

We hypothesized that greater post-stroke fatigue would be associated with altered resting-state functional connectivity of bilateral dorsolateral prefrontal, caudal anterior cingulate, precuneus, insular, and thalamic regions, particularly within frontoparietal, limbic, and thalamocortical networks involved in cognitive control, motivational processing, interoception, and arousal after accounting for depressive symptoms.

## 2 Methods

### 2.1 Participants and behavioral measures

Participants included individuals with stroke and age-matched control participants enrolled in a longitudinal neuroimaging study carried out at Washington University in Saint Louis (Corbetta et al., 2015). Stroke participants were assessed at acute (0-month), 3-month, and/or 12-month timepoints; controls were assessed at two timepoints (0-month, 3-month). All participants provided informed consent under an institutional review board-approved protocol. Controls were healthy age-, sex- and education-matched adults (demographic/cognitive differences that could influence questionnaire performance and neuroimaging measures). Stroke participants were enrolled within two weeks of first symptomatic ischemic or hemorrhagic stroke, with clinical evidence of motor, language, attention, visual, or memory deficits. Key exclusion criteria included previous stroke, multifocal strokes, and significant comorbid neurological or psychiatric conditions that could confound imaging or behavioral interpretation (Corbetta et al., 2015). The final analytic sample comprised participants with complete resting-state fMRI data, vitality scores, and depressive symptom ratings across at least one study timepoint (Fig. 1).

**Fig. 1:**
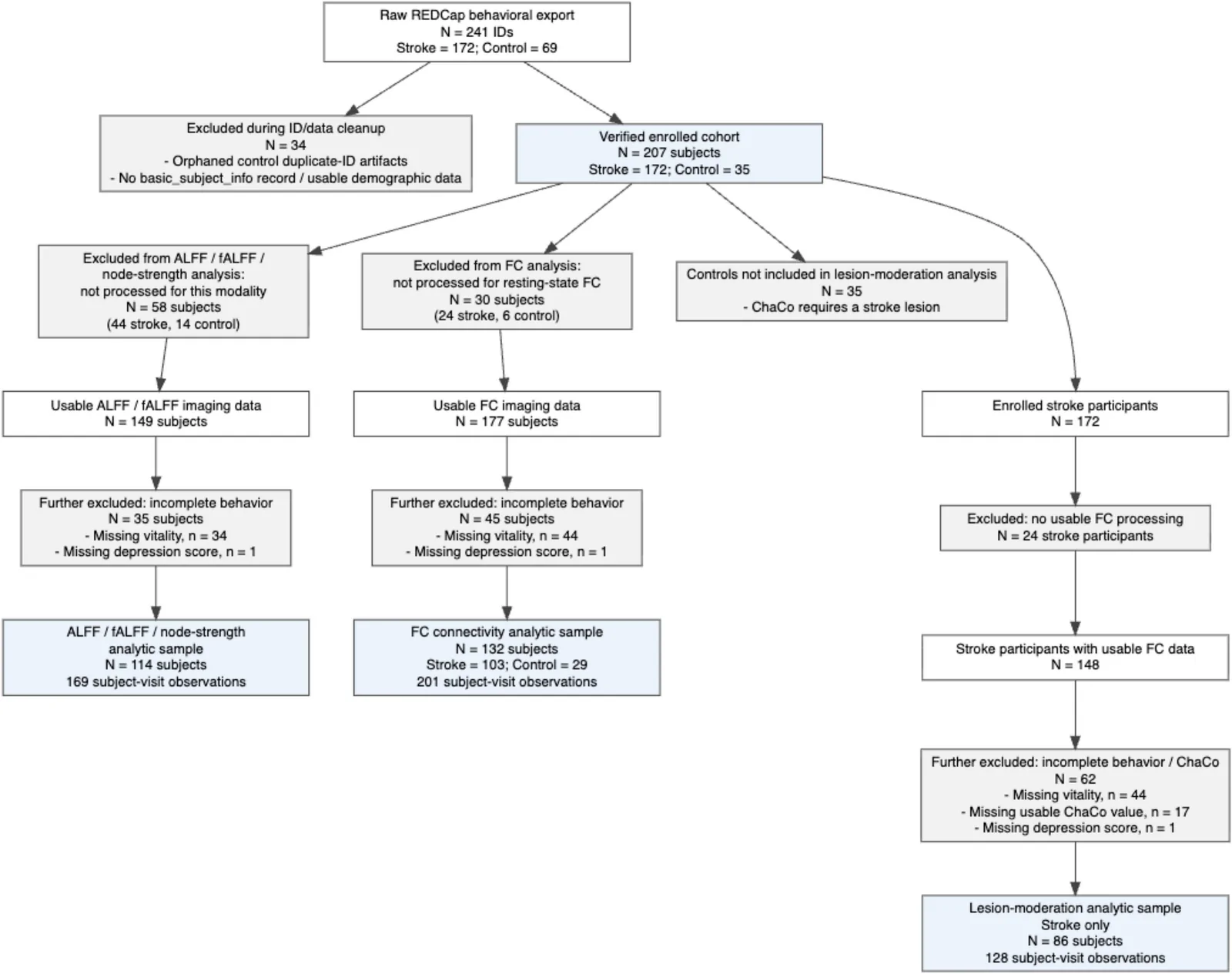
Participant flow diagram. Participant accounting begins with the enrolled cohort that could be directly verified from the present dataset; recruitment and screening procedures for the parent longitudinal cohort are described previously by Corbetta et al. (2015). Subsequent exclusions and analytic sample sizes were derived directly from the current behavioral and imaging datasets. Final analytic samples comprised 114 participants (169 subject-visit observations) for ALFF/fALFF and node-strength analyses, 132 participants (103 stroke, 29 controls; 201 subject-visit observations) for functional connectivity analyses, and 86 stroke participants (128 subject-visit observations) for the lesion-moderation analysis.

Fatigue was assessed using the SF-36 Vitality (VT) subscale, a continuous measure reflecting energy and fatigue, with lower scores indicating higher fatigue/reduced vitality. Depressive symptoms were assessed using the Geriatric Depression Scale - Short (GDS-15) a 15-item scale, treated as a continuous variable. Because vitality and depressive symptoms are conceptually related, their association was quantified at the subject level using Pearson and Spearman correlations. Multicollinearity was assessed using variance inflation factors (VIFs). Because inclusion of interaction terms can increase collinearity between predictors and their interaction terms, VIFs were examined for the fitted models. Where elevated VIFs were observed, sensitivity analyses using mean-centered continuous predictors were performed to assess the stability of coefficient estimates and statistical inference.

### 2.2 fMRI acquisition, preprocessing, and postprocessing

Imaging data were acquired on a Siemens 3 T Tim-Trio scanner using a 12-channel head coil. Resting-state fMRI was collected using gradient-echo echo-planar imaging (TR = 2000 ms, TE = 27 ms, 32 axial slices, 4 mm thickness, 4 × 4 mm in-plane resolution). Participants fixated on a central cross during scanning. Six to eight runs were acquired per participant (128 volumes per run; ∼30 min total).

Data were preprocessed using fMRIPrep (v23.2.1) (Esteban et al., 2019). Anatomical images were bias-corrected using N4BiasFieldCorrection (Tustison et al., 2010), skull-stripped, segmented using FAST (Zhang et al., 2001), and cortical surfaces were reconstructed with FreeSurfer (v7.3.2) (Dale et al., 1999). Functional images were corrected for head motion using mcflirt (Jenkinson et al., 2002) and susceptibility distortions (Wang et al., 2017), co-registered to T1-weighted images using boundary-based registration (Greve and Fischl, 2009), and normalized to MNI152NLin6Asym space (Evans et al., 2012) via Advanced Normalization Tools (ANTs) (Avants et al., 2008).

Postprocessing was performed using ICA-AROMA (fMRIPost-AROMA v0.0.12 (Pruim et al., 2015). Data were spatially smoothed (6 mm FWHM) (Smith and Brady, 1997) and motion-related components were removed. Global signal regression was included. Both pipelines used Nipype for workflow management (Gorgolewski et al., 2011) and Nilearn for internal operations (Abraham et al., 2014). Runs underwent automated quality control based on framewise displacement (Power et al., 2014) and DVARS thresholds, and participants were required to have ≥4 valid runs for inclusion.

### 2.3 Functional connectivity analysis

Resting-state functional connectivity (FC) was computed using region- and network-level approaches from denoised BOLD timeseries. Regional FC was computed using the fs86 anatomical parcellation derived from FreeSurfer segmentation, comprising 86 cortical and subcortical regions. Mean BOLD time series were extracted from each parcel, and pairwise Pearson correlation coefficients were calculated between regional time series. Functional networks were defined by mapping fs86 parcels to canonical large-scale Yeo network assignments (e.g., Ventral Attention Network, Default Mode Network) (Thomas Yeo et al., 2011). Connectivity metrics included whole-brain mean connectivity, within-network connectivity (Yeo-7 networks), between-network connectivity, and ROI-based connectivity.

Specific regional FC analyses were performed using predefined cortical and subcortical regions, including the dorsolateral prefrontal cortex (DLPFC; rostral middle frontal), caudal anterior cingulate cortex (Caudal ACC), precuneus, insula, and thalamus. Regions were selected based on their established roles in fatigue-relevant neural processes. The dorsolateral prefrontal cortex (DLPFC; rostral middle frontal gyrus) was selected for its role in cognitive control and effort allocation (i.e., top-down regulation of effortful behavior) (Friedman and Robbins, 2022; Miller and Cohen, 2001); the caudal anterior cingulate cortex (caudal ACC) for effort monitoring and motivational processing (Shenhav et al., 2013); the precuneus as a hub of the default mode network (DMN) implicated in self-referential processing and mental fatigue (Grossner et al., 2019; Ren et al., 2024); the insula for interoceptive processing and bodily state awareness (i.e., internal energy representation) (Avery et al., 2014); and the thalamus for arousal regulation and interoceptive relay via thalamocortical circuits (Shine et al., 2016). Each region was defined bilaterally using the mean of left and right parcels. Region-to-network connectivity was defined as the mean correlation between a region of interest and all parcels assigned to a target network. ROI-to-ROI connectivity was defined as the mean pairwise correlation across all ROI combinations between two ROI sets, accounting for bilaterally defined regions.

Connectivity values were Fisher z-transformed prior to statistical modeling to stabilize variance and approximate normality.

### 2.4 Regional amplitude analysis (ALFF/fALFF)

Regional neural signal amplitude was quantified using amplitude of low-frequency fluctuations (ALFF) and fractional ALFF (fALFF) derived from the same fs86 parcellation. ALFF and fALFF values were computed from resting-state time series after preprocessing and summarized at both the regional (ROI-level) and large-scale network levels by averaging parcels belonging to canonical functional networks. Network-level and ROI-level amplitude measures were analyzed as separate feature families to account for differences in spatial scale and statistical dimensionality. Because ALFF/fALFF quantifies regional signal amplitude rather than inter-regional coupling, these analyses were interpreted as complementary tests of local neural activity rather than circuit-level connectivity effects.

### 2.5 Structural disconnection (ChaCo)

ChaCo (Change in Connectivity) scores were computed using the Network Modification (NeMo) Tool (Kuceyeski et al., 2013). Each patient's lesion mask was put into the NeMo tool, which references a normative tractography database derived from diffusion MRI acquired in healthy controls; no diffusion or other advanced imaging was acquired from our own patients for this estimate. For each edge in the structural connectome, NeMo estimates the percentage of the normative white-matter streamlines connecting that edge's two endpoints that pass through the lesion mask, yielding a per-edge disconnection score between 0 (no disruption) and 1 (complete estimated disconnection). ChaCo is therefore a lesion-based estimate of structural disconnection, not a direct measurement of the patient's own white-matter tracts. ChaCo measures were treated as subject-level indices of structural disconnection (Kuceyeski et al., 2013). They were treated as time-invariant after stroke predictors and linked to longitudinal behavioral and imaging measures within stroke-only moderation models.

### 2.6 Statistical modeling framework

Associations between behavioral measures and imaging features were examined using linear mixed-effects models implemented in R (lme4 package). Random intercepts were specified for subject to account for repeated observations across timepoints. Time was modeled as months post-event when available. Models were estimated using maximum likelihood (REML = FALSE) to allow comparison across nested specifications.

### 2.7 Whole-brain analyses

Initial exploratory models tested vitality-functional connectivity associations across whole-brain FC feature families. To determine whether imaging feature-vitality associations were independent of depressive burden, models included GDS-15 as a covariate.

For each connectivity feature, the following base model was estimated:

$$\mathrm{Imaging Featur}e_{\mathrm{ij}}=\beta_{0}+\beta_{1}\;\mathrm{Vitalit}y_{\mathrm{ij}}+\beta_{2}\mathrm{GDS}S_{\mathrm{ij}}+\beta_{3}\;\mathrm{Grou}p_{i}+\beta_{4}\;{\left(\mathrm{Vitality}\;x\;\mathrm{Group}\right)}_{\mathrm{ij}}+\beta_{5}\;\mathrm{Ag}e_{i}+\beta_{6}\;\mathrm{Se}x_{i}+\beta_{7}\;\mathrm{Tim}e_{\mathrm{ij}}+u_{i}+\varepsilon_{\mathrm{ij}.}$$

Where (*i*) indexes participant, (*j*) indexes timepoint, $u_{i}$ represents the subject-specific random intercept and $\varepsilon_{\mathrm{ij}}$ the residual error term.

This specification allowed simultaneous estimation of vitality- and depression-related effects while controlling for shared variance.

### 2.8 Residualized vitality sensitivity analysis

As a complementary approach to isolate vitality-specific variance, vitality was residualized with respect to depressive symptoms and covariates:

*Vitality*_*resid*_ *= residuals (Vitality ∼ GDS-15 + Age + Sex + Group + Time)*

Residualized vitality was then substituted into the mixed-effects models. This analysis isolates variance in vitality that is statistically independent of depressive symptoms. To assess whether item-level overlap between depressive symptoms and vitality influenced the residualized analyses, we performed a sensitivity analysis in which the overlapping energy/anergia item was removed from the GDS-15 before residualizing SF-36 Vitality scores. The validated four-item SF-36 Vitality scale was retained intact. The residualized connectivity models were then repeated using this item-reduced depression score.

### 2.9 Group difference analyses

Group differences in ALFF, fALFF, and functional connectivity node strength were assessed using mixed-effects models with GDS-15 and covariates included. Parcel-wise t-statistics for the group effect were visualized on cortical and subcortical surfaces using the Desikan-Killiany atlas. These analyses were conducted to characterize broader stroke-related neural differences and were considered exploratory.

### 2.10 Model diagnostics and significance testing

Model convergence was assessed using the bobyqa optimizer with extended iteration limits when required. Fixed-effect estimates, standard errors, test statistics, and *p*-values were extracted. Marginal (R^2^m, variance explained by fixed effects alone) and conditional (R^2^c, variance explained by fixed and random effects together) R-squared are reported (Nakagawa and Schielzeth, 2013) for each key mixed model. Multicollinearity was assessed using VIFs. False discovery rate (FDR) correction was applied using the Benjamini-Hochberg procedure within predefined feature families (e.g., FC network-level, ROI-based connectivity, regional amplitude). These families were defined a priori based on modality and spatial scale.

### 2.11 Stroke-only structural disconnection moderation

To test whether structural disconnection severity modulated vitality-imaging feature relationships in stroke, stroke-only moderation models were fit. Vitality, ChaCo, and GDS-15 were z-scored within the stroke sample prior to modeling to facilitate interpretation of interaction terms. Depressive symptoms (GDS-15) were included as a covariate to ensure that any moderation effect reflected fatigue-specific rather than depression-related variance. The following model was estimated for each connectivity feature:

$${\mathrm{Imaging Feature}}_{\mathrm{ij}}=\beta_{0}+\beta_{1}\;{\mathrm{Vitality}}_{\mathrm{ij}}\_z+\beta_{2}{\mathrm{ChaCo}}_{\mathrm{ij}}\_z+\beta_{3}\;\;{\mathrm{GDSS}}_{\mathrm{ij}}\_z+\beta_{4}\left({\mathrm{Vitality}}_{\mathrm{ij}}\_z\;x\;{\mathrm{ChaCo}}_{i}\_z\right)+\beta_{5}\;{\mathrm{Age}}_{i}+\beta_{6}\;{\mathrm{Sex}}_{i}+\beta_{7}\;{\mathrm{Time}}_{\mathrm{ij}}+u_{i}+\varepsilon_{\mathrm{ij}}$$

The primary test of interest was the Vitality_z × ChaCo_z interaction term, reflecting whether the fatigue-connectivity relationship varied as a function of structural disconnection severity after accounting for depressive burden. ChaCo values themselves were not modeled as an outcome. FDR correction was applied within each model family across tested endpoints. Where models failed to converge with a random slope for vitality, a random intercept-only specification was used.

## 3 Results

### 3.1 Participants

The final analytic sample comprised 132 participants (103 stroke, 29 controls; Table 1). Stroke participants had a mean age of 54.1 years (SD = 9.9) and controls had a mean age of 55.2 years (SD = 12.0); groups did not differ significantly in age (Wilcoxon *p* = 0.636). The sample was 49.2% female and 50.8% male, with no significant sex difference between groups (χ^2^ *p* = 0.608). The majority of participants identified as Black or African American (65.2%), with 34.8% identifying as White and 3.0% as Hispanic or Latino. Education was comparable across groups (stroke: 13.2 ± 2.5 years; control: 13.6 ± 2.7 years; Wilcoxon *p* = 0.681).

**Table 1:** Descriptive data.

Variable	Stroke	Control	Total
DEMOGRAPHICS			
N	103	29	132
Age mean (SD)	54.1 (9.9)	55.2 (12.0)	54.3 (10.4)
Age median [IQR]	54.0 [50.0–60.0]	54.0 [51.0–60.0]	54.0 [50.0–60.0]
Female n (%)	49 (47.6%)	16 (55.2%)	65 (49.2%)
Male n (%)	54 (52.4%)	13 (44.8%)	67 (50.8%)
White n (%)	35 (34.0%)	11 (37.9%)	46 (34.8%)
Black or African American n (%)	68 (66.0%)	18 (62.1%)	86 (65.2%)
Hispanic or Latino n (%)	2 (1.9%)	2 (6.9%)	4 (3.0%)
Right-handed n (%)	96 (93.2%)	27 (93.1%)	123 (93.2%)
Education years mean (SD)	13.2 (2.5)	13.6 (2.7)	13.3 (2.6)
OUTCOME METRICS			
Vitality mean (SD)	54.4 (23.7)	67.3 (21.5)	57.2 (23.8)
Vitality median [IQR]	55.0 [38.8–70.0]	75.0 [65.0–80.0]	65.0 [40.0–75.6]
GDS-15 mean (SD)	4.1 (3.8)	2.3 (3.2)	3.7 (3.8)
GDS-15 median [IQR]	2.5 [1.0–6.8]	1.0 [0.5–3.5]	2.2 [0.5–6.0]
1 timepoint n (%)	21 (20.4%)	0 (0.0%)	21 (15.9%)
2 timepoints n (%)	82 (79.6%)	29 (100.0%)	111 (84.1%)
3 timepoints n (%)	0 (0.0%)	0 (0.0%)	0 (0.0%)
NIHSS median [IQR]	4.5 [2.0–9.0]	–	–
LESION CHARACTERISTICS			
Lesion size mm3 median [IQR]	1278.0 [325.8–5172.8]	–	–
Left hemisphere n (%)	54 (52.4%)	–	–
Right hemisphere n (%)	49 (47.6%)	–	–
Bilateral n (%)	0 (0.0%)	–	–
Ischemic n (%)	81 (78.6%)	–	–
Hemorrhagic n (%)	17 (16.5%)	–	–
Lesion type: Missing n (%)	5 (4.9%)	–	–
Lesion Site			
*Subcortical n (%)*	17 (16.5%)	–	–
*Cortical n (%)*	27 (26.2%)	–	–
*Cortico-subcortical n (%)*	17 (16.5%)	–	–
*White matter only n (%)*	6 (5.8%)	–	–
*Brainstem n (%)*	12 (11.7%)	–	–
*Cerebellar n (%)*	6 (5.8%)	–	–
*Other n (%)*	1 (1.0%)	–	–
*Missing n (%)*	17 (16.5%)	–	–
TREATMENT			
tPA n (%)	13 (12.6%)	–	–
Thrombectomy n (%)	0 (0.0%)	–	–

Among stroke participants, the majority had ischemic stroke (78.6%), with 16.5% hemorrhagic and 4.9% with missing lesion type data. Median NIHSS at hospital admission was 4.5 [IQR: 2.0–9.0], indicating mild to moderate stroke severity. Lesions were distributed across the left (52.4%) and right (47.6%) hemispheres. By lesion site, the most common locations were cortical (26.2%), subcortical (16.5%), and cortico-subcortical (16.5%), with brainstem (11.7%), white matter only (5.8%), cerebellar (5.8%), and other (1.0%) representing smaller proportions; 16.5% were missing lesion site data. Median lesion size was 1278.0 mm^3^ [IQR: 325.8–5172.8]. Thirteen stroke participants (12.6%) received tPA.

Stroke participants reported greater fatigue than controls (vitality mean: 54.4 vs. 67.3; Wilcoxon *p* = 0.004) and higher depressive symptom burden (GDS-15 mean: 4.1 vs. 2.3; Wilcoxon *p* = 0.004). Vitality and depressive symptoms were moderately correlated at the subject level (Pearson *r* = −0.742, Spearman *r* = −0.724, both *p* < 0.001), consistent with shared but non-redundant variance between constructs. Multicollinearity was assessed using variance inflation factors (VIFs) calculated from the fixed-effects design matrix. Because models included a vitality × group interaction, vitality was mean-centered before constructing the interaction term. In the functional connectivity models, mean-centering reduced the VIFs for group and the vitality × group interaction from 8.82 and 10.39 to 1.48 and 4.90, respectively. The VIF for vitality remained 6.52, whereas VIFs for depressive symptoms and other covariates were < 2.0. Mean-centering did not alter the estimated vitality or vitality × group interaction effects. The majority of stroke participants contributed two timepoints (79.6%), with 20.4% contributing one timepoint; all control participants contributed two timepoints. Full demographic and clinical characteristics are presented in Table 1.

### 3.2 Linear mixed effects models

Depression-adjusted models revealed that greater vitality (less fatigue) was associated with increased frontoparietal-limbic (FP-LIM) network connectivity (β = 0.0013, FDR q = 0.048, *p* = 0.001, FP-LIM, R^2^m = 0.075, R^2^c = 0.444, *n* = 201, 132 subjects; Fig. 2). This was the only network-level connectivity pair to survive FDR correction, and depressive symptom severity was not independently associated with FP-LIM connectivity in the same model (*p* = 0.197). However, this association did not survive FDR correction in the residualized vitality model (FDR q = 0.389), suggesting that the FP-LIM effect depends at least partly on variance shared between fatigue and depression rather than reflecting purely fatigue-specific neural coupling. Four additional network pairs showed nominal associations (uncorrected *p* < 0.05), including limbic connectivity with somatomotor and subcortical networks and DMN-visual connectivity, but none survived multiple comparisons correction (Fig. 2).

**Fig. 2:**
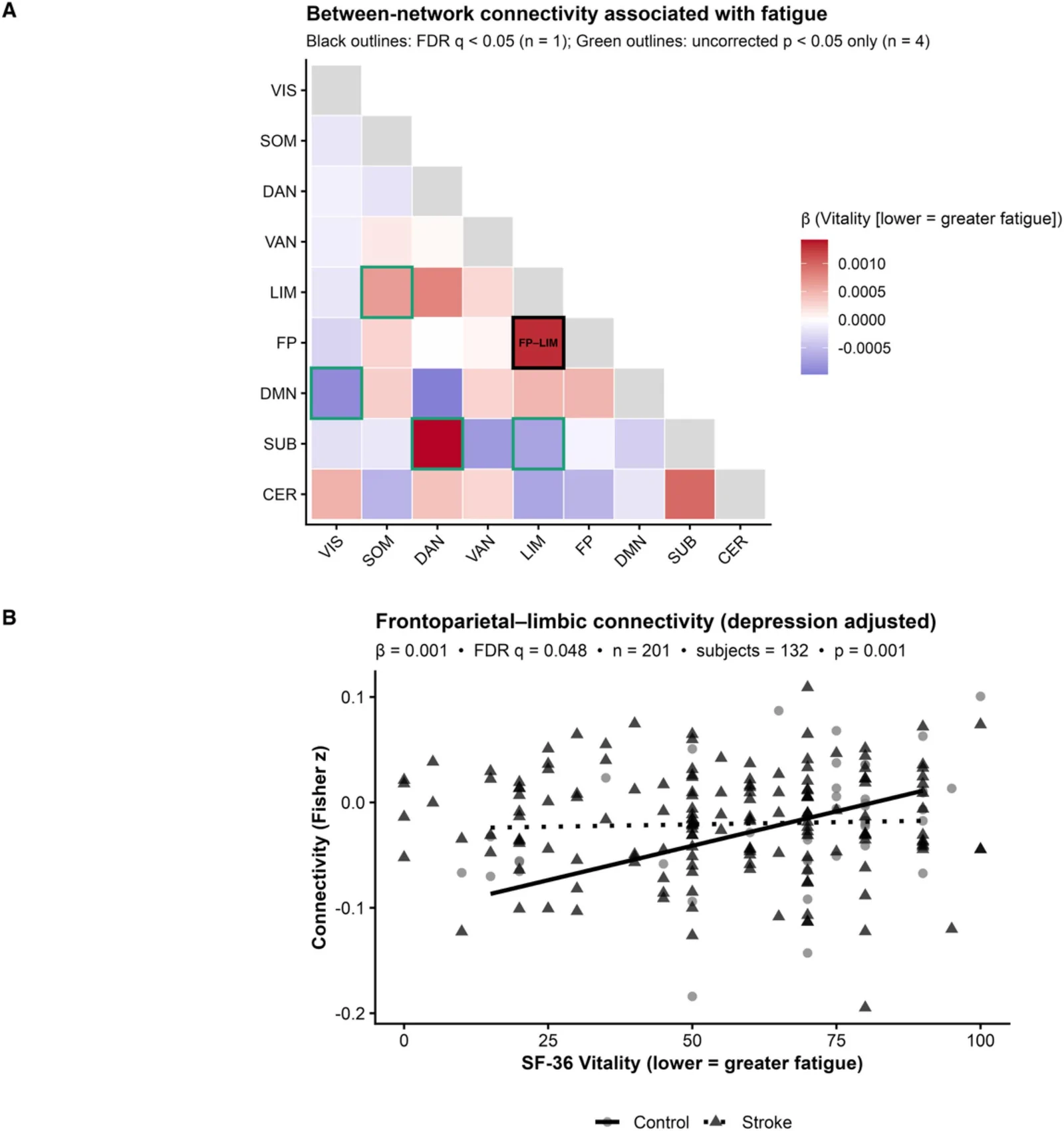
Between-network connectivity associated with fatigue. (A) Lower triangular matrix displaying β coefficients for the vitality predictor (SF-36 Vitality; lower = greater fatigue) from depression-adjusted mixed-effects models across all between-network connectivity pairs. Color reflects direction and magnitude of association (red = positive; blue = negative). Black outline indicates the single pair surviving FDR correction in the depression-adjusted model (FP-LIM, q = 0.048); green outlines indicate pairs significant at uncorrected *p* < 0.05 only (*n* = 4). Gray cells = non-significant. Networks: VIS = Visual; SOM = Somatomotor; DAN = Dorsal Attention; VAN = Ventral Attention; LIM = Limbic; FP = Frontoparietal; DMN = Default Mode; SUB = Subcortical; CER = Cerebellar. (B) FP-LIM between-network connectivity as a function of SF-36 Vitality score, adjusted for depressive symptoms and covariates (β = 0.0013, FDR q = 0.048, *p* = 0.001). Regression lines reflect fixed-effects slopes for controls (solid) and stroke participants (dashed). Note this association did not survive FDR correction in the residualized vitality model (FDR q = 0.389), indicating partial overlap with depression-related variance.

Whole brain, within network and ROI-based functional connectivity models did not demonstrate any FDR significant results; nominally significant results (*p* < 0.05) can be found in Supplementary Table 1(Table S1). Stroke only structural disconnection moderation analyses did not show any FDR significant results (Table S3)

### 3.3 Residualized mixed effect models

To isolate fatigue-specific neural associations from depressive burden, vitality scores were residualized with respect to GDS-15 and covariates prior to modeling. The only residualized vitality scores to survive FDR correction were increased thalamus-insula connectivity (β = −0.0071, FDR q = 0.011, *p* < 0.001, R^2^m = 0.066, R^2^c = 0.352, *n* = 201, 132 subjects; Fig. 3A) and increased thalamus-ventral attention network (VAN) connectivity (β = −0.0043, FDR q = 0.012, *p* < 0.001, R^2^m = 0.124, R^2^c = 0.503; Fig. 3B) across the full sample. Vitality × group (stroke) interactions were nominally significant (thalamus-insla β = 0.0029, FDR q = 0.653, *p* = 0.014; thalamus-VAN β = 0.0022, FDR q = 0.653, *p* = 0.018). Because vitality and stroke-versus-control status are correlated in this sample (variance inflation factor = 6.52 for vitality in the FC family, stable whether or not the interaction term is centered), these models cannot fully separate a vitality-specific effect from a stroke-status effect. The remaining nominally significant residualized results can be found in Supplementary Table 2 (Table S2).

**Fig. 3:**
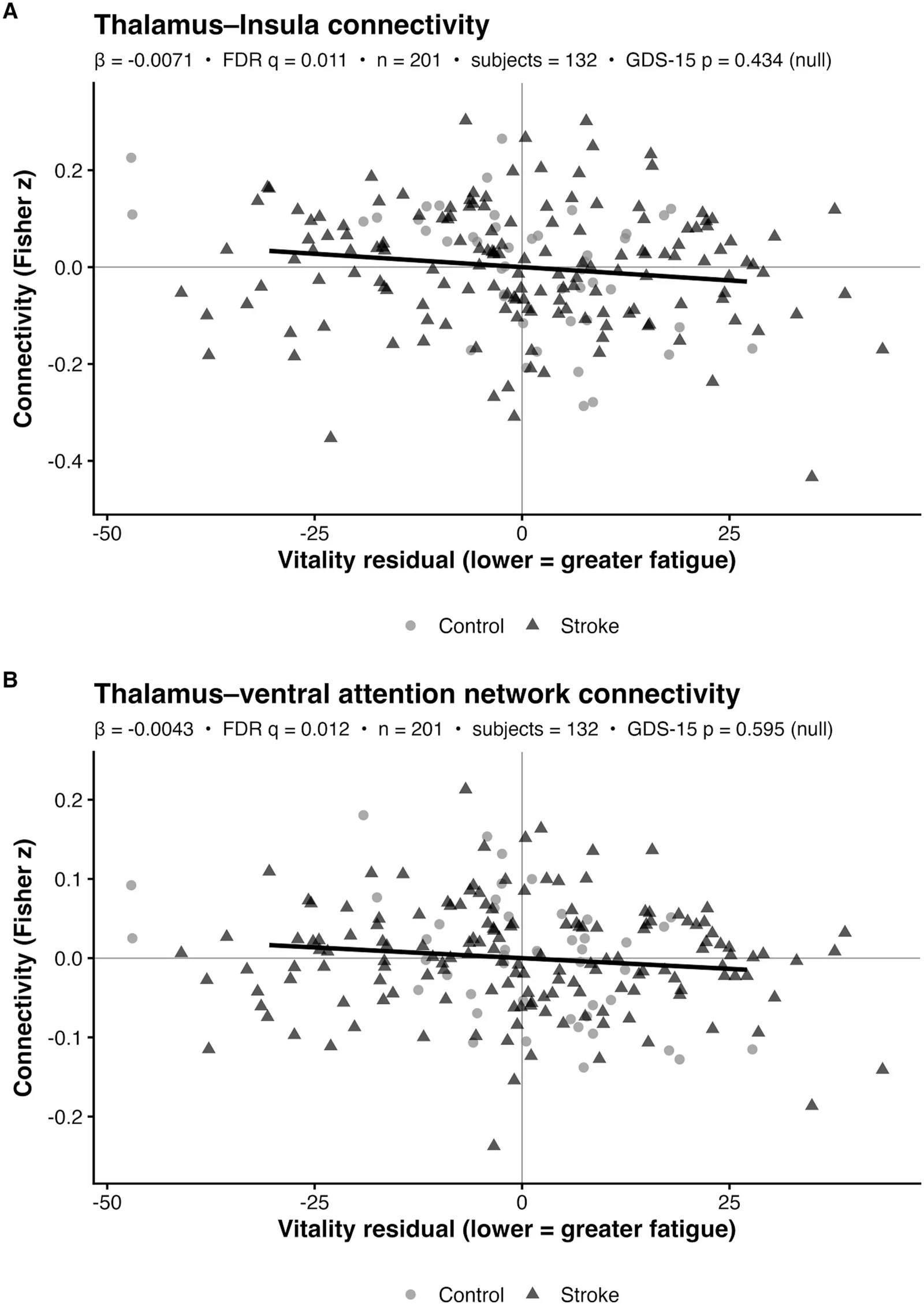
Associations between residualized vitality and thalamic connectivity. Scatter plots show functional connectivity as a function of residualized vitality for thalamus-insula (A) and thalamus-ventral attention network (B) circuits. Vitality residuals were obtained by regressing SF-36 Vitality scores on depressive symptoms (GDS-15), age, sex, group, and time post-event. Regression lines reflect fixed effects from linear mixed-effects models with random intercepts for subject. Header statistics (β, FDR q) reflect the residualized vitality mixed-effects model. GDS-15 *p*-values reflect the association of depressive symptoms with connectivity in the same depression-adjusted model, demonstrating the absence of a depression effect. Thalamus-insula, R2m = 0.066, R2c = 0.352; thalamus-VAN, R2m = 0.124, R2c = 0.503. Circles = controls; triangles = stroke participants.

Results were largely unchanged and remained significant after FDR correction after removing the overlapping energy/anergia item from the depression measure (GDS-15_13; “Do you feel full of energy”). The thalamic associations were minimally attenuated and remained significant after FDR correction (thalamus-insula: β = −0.0066, q = 0.016; thalamus-VAN: β = −0.0041, q = 0.016), compared with the original residualized models (β = −0.0071, q = 0.011 and β = −0.0043, q = 0.012, respectively).

As a sensitivity check for motion, mean framewise displacement (FD) was computed per scan session and averaged per subject. Mean FD was available for 114 participants (93 stroke, 21 controls). FD did not differ significantly by group (Control: mean 0.282, SD 0.096; Stroke: mean 0.276, SD 0.084; Wilcoxon W = 1000, *p* = 0.866.We then re-fit both key thalamic models (thalamus-insula, thalamus-VAN) with subject-level mean FD added as a covariate. FD was not a significant predictor of either connectivity metric (thalamus-insula FD mean: β = 0.108, *t* = 0.615; thalamus-VAN: β = 0.074, *t* = 0.668), and the vitality and vitality × group interaction terms of particular interest were stable in both direction and magnitude with FD included. For the vitality main effect, thalamus-insula β = −0.00422, *t* = −2.99 without FD vs. β = −0.00444, *t* = −2.63 with FD, and thalamus-VAN β = −0.00289, *t* = −3.13 vs. β = −0.00306, *t* = −2.84; for the interaction thalamus-VAN Vitality × Group: β = 0.00224 without FD vs. 0.00257 with FD, thalamus-insula Vitality × Group: β = 0.00291 vs. 0.00308. Together, this provides no evidence that the thalamic associations were driven by between-group differences in head motion. We also re-fit both models on GSR-off connectivity estimates for the same subjects (*n* = 201 observations, 100% matched). The direction of both the vitality effect and the vitality × group interaction was preserved under GSR-off, but neither retained statistical significance (thalamus-VAN: vitality effect *t* = −3.13 under GSR-on vs. *t* = −1.58 under GSR-off; interaction *t* = 2.41 vs. *t* = 0.62; thalamus-insula: vitality effect *t* = −2.99 vs. *t* = −1.56; interaction *t* = 2.06 vs. *t* = 0.52).

To test whether fatigue-related thalamic hyperconnectivity varied as a function of stroke lesion location, lesion site was entered as a moderator in stroke-only mixed-effects models. Lesion site was categorized as subcortical, cortical, mixed cortico-subcortical, or posterior/other (brainstem, cerebellar, other). The residualized vitality × lesion site interaction was non-significant for all site categories for both thalamus-insula (cortical: *p* = 0.693; mixed: *p* = 0.500; posterior/other: *p* = 0.595) and thalamus-VAN (cortical: *p* = 0.417; mixed: *p* = 0.197; posterior/other: *p* = 0.698), indicating that fatigue-related thalamic hyperconnectivity did not differ across lesion site categories. This pattern held regardless of whether lesions directly involved subcortical or thalamic structures.

Similarly, lesion volume did not moderate the fatigue-thalamic connectivity relationship. The vitality × lesion size interaction was non-significant for both thalamus-insula (β = 0.0005, *p* = 0.610) and thalamus-VAN (β = 0.0006, *p* = 0.276), indicating that the effect was consistent regardless of lesion burden.

### 3.4 Functional connectivity regional signal amplitude and node strength

To characterize the broader pattern of neural disruption in stroke, group differences in regional signal amplitude and whole-brain functional connectivity node strength were examined between stroke and control participants using depression-adjusted mixed-effects models (Supplementary Tables S4, S5, Fig. S1). FC node strength was reduced in stroke in 5 regions, including left insula (*t* = −2.68, *p* = 0.007), left rostral anterior cingulate cortex (*t* = −2.31, *p* = 0.021), left nucleus accumbens (*t* = −2.31, *p* = 0.021), right superior frontal cortex (*t* = −2.17, *p* = 0.030), and right medial orbitofrontal cortex (*t* = −2.11, *p* = 0.035). None of these group differences survived FDR correction and should be interpreted as exploratory (Fig. 4).

**Fig. 4:**
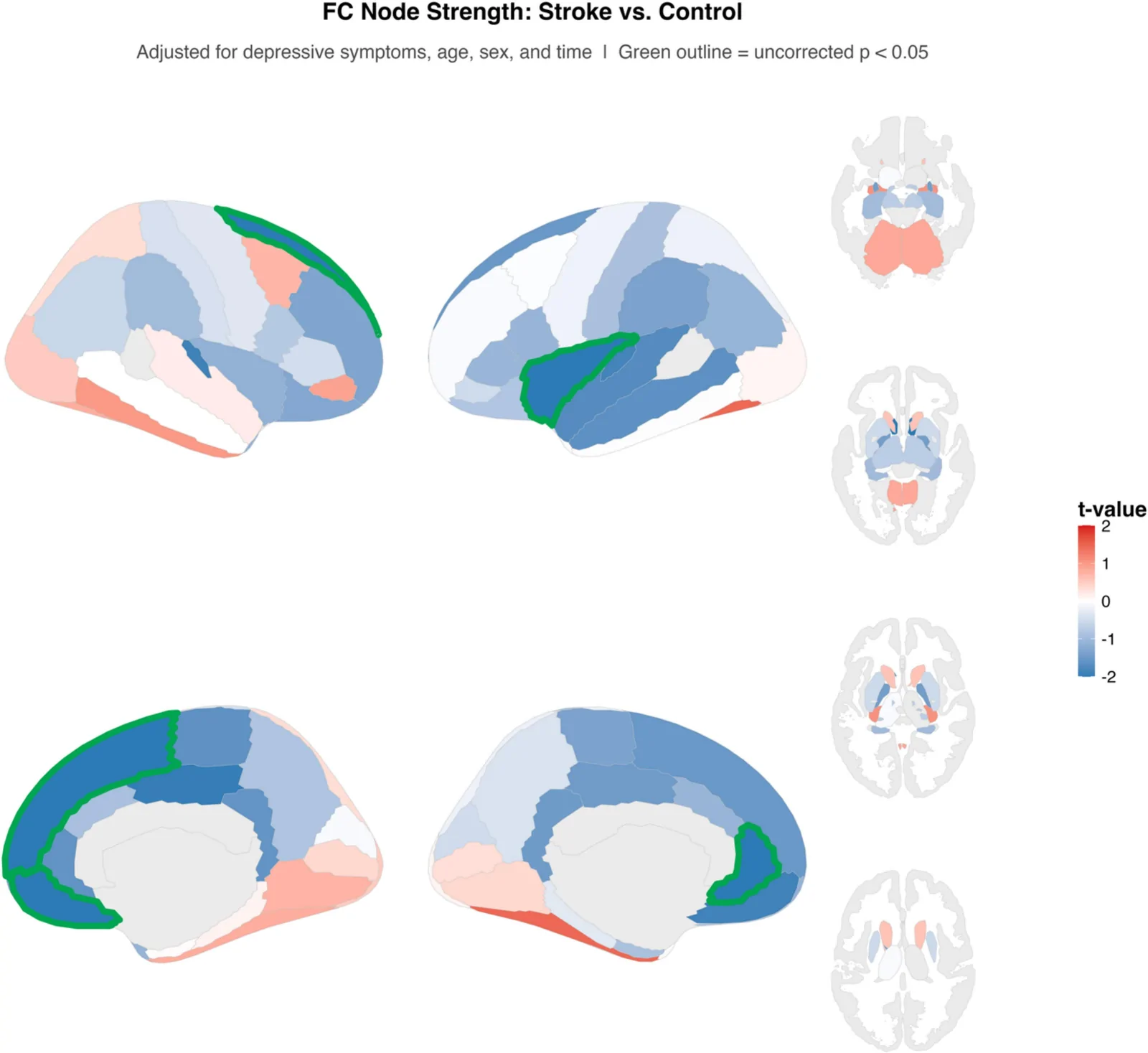
Stroke-related differences in regional connectivity node strength. Surface maps display t-values for the stroke vs. control group contrast from depression-adjusted mixed-effects models (GDS-15 covariate included), shown for FC Node Strength. Top row = lateral surface views; bottom row = medial surface views. Subcortical axial slices are shown to the right of each cortical panel. Warm colors (red) indicate greater values in stroke relative to controls; cool colors (blue) indicate reduced values. Color scale reflects t-values. Green outlines denote regions with uncorrected *p* < 0.05; no regions survived FDR correction. FC Node Strength showed nominally significant reductions in stroke in 5 regions including left insula, left rostral anterior cingulate, left nucleus accumbens, right superior frontal cortex, and right medial orbitofrontal cortex. These analyses were conducted separately from the vitality analyses in Fig. 1, Fig. 2 and characterize the broader pattern of stroke-related neural disruption rather than fatigue-specific effects.FC = functional connectivity.

## 4 Discussion

Post-stroke fatigue is among the most prevalent and disabling sequelae of stroke, yet its neural substrates remain poorly understood and its frequent conflation with depression has limited both mechanistic research and clinical trial design (Cumming et al., 2016; Jolly et al., 2023; Paciaroni and Acciarresi, 2019). The present study examined resting-state neuroimaging correlates of fatigue in a longitudinal cohort of stroke subjects and controls, using within- and between-network connectivity and regional signal amplitude. Our primary aim was to separate fatigue-related neural signals from co-occurring depressive burden. Our findings point to two partially separable neural correlates of fatigue and suggest a broader pattern of neural disruption that may provide context for these circuit-level effects.

### 4.1 Frontoparietal-limbic connectivity and fatigue

Greater fatigue was associated with reduced frontoparietal-limbic (FP-LIM) between-network connectivity in the depression-adjusted models, and this was the only network-level finding to survive FDR correction across the full sample (Fig. 2). The frontoparietal network is thought to play a role in cognitive control, effort allocation, and goal-directed behavior (i.e., top-down regulation of behavior) (Kuppuswamy et al., 2024; Ondobaka et al., 2022), while limbic circuitry is involved in motivational and affective processing (Kim and Hamann, 2007; Towfighi et al., 2017). Reduced coupling between these systems is consistent with models of fatigue as a failure of top-down motivational regulation, in which altered frontoparietal modulation of limbic structures may impair the ability to initiate and sustain effortful behavior (Cotter et al., 2022). Of note, this association was present across both stroke and control participants without a significant group interaction, suggesting that FP-LIM connectivity may reflect a general neural correlate of fatigue rather than a stroke-specific mechanism.

However, the FP-LIM association did not survive FDR correction in the residualized vitality model. This suggests that FP-LIM connectivity may be more sensitive to variance shared between fatigue and depression than to fatigue-specific neural burden, and may index a broader dimension of affective and motivational dysregulation common to both conditions rather than a substrate unique to PSF (Lassalle-Lagadec et al., 2012; Zhang et al., 2021). Clinically, this does not exclude a role for frontoparietal neuromodulation. Targeting DLPFC or ACC circuits may improve fatigue by enhancing cognitive control, effort regulation, or mood-related processes, even in patients without depression, although the mechanisms and magnitude of benefit may differ from those with comorbid depression.

### 4.2 Thalamic connectivity shows a partially dissociable association with fatigue

In contrast, thalamic hyperconnectivity emerged as a more fatigue-specific finding (Fig. 2). Greater fatigue was associated with increased thalamus-insula and thalamus-ventral attention network (VAN) connectivity in the residualized vitality model for the whole sample. Depressive symptom severity was not independently associated with thalamic connectivity in either circuit, and effects persisted after removing variance shared between fatigue and depression. This pattern suggests that thalamic hyperconnectivity may track a neurobiological component of fatigue that is distinct from depressive burden. However, the nominally significant vitality × group interaction indicates that this relationship was stronger in controls than in stroke participants. This pattern may reflect stroke-related disruption of normal thalamocortical fatigue signaling, where stroke pathology may attenuate or reorganize neural architecture, such that the thalamic fatigue signal observed in healthy individuals is diminished or masked in stroke survivors. Alternatively, the full-sample effect may reflect shared physiological variation in fatigue and thalamic connectivity that operates similarly across healthy and stroke populations at the group level, even if it is not detectable within the stroke subgroup alone. In addition, fatigue-related thalamic hyperconnectivity did not vary as a function of lesion site or lesion volume, consistent with a potential functional network contribution to PSF (Boot et al., 2022; Jolly et al., 2023), though the lesion-site subgroups were small (*n* = 19–40) and the moderation analyses should be considered preliminary.

The thalamus is thought to play a central role in arousal regulation, sensory gating, and interoceptive signaling, with connections to both the insula and attentional networks (Killgore et al., 2015; Portas et al., 1998; Shine et al., 2016). Increased thalamo-insular connectivity may reflect impaired relay of bodily state information to insular cortex (Craig, 2009; Shine et al., 2016), which is thought to be important for representing energy availability and internal homeostatic demand (Wu et al., 2015b). Disruption of this pathway could contribute to the subjective experience of persistent exhaustion that characterizes PSF. Thalamo-VAN hyperconnectivity may complement this finding, as the VAN is thought to support stimulus-driven attentional reorienting (Corbetta and Shulman, 2002), and increased thalamic input to this network could reflect impaired arousal-driven engagement with the environment, a feature commonly reported by patients with PSF (Paciaroni and Acciarresi, 2019; Wu et al., 2015b). Together, our data suggest that increased thalamic connectivity with both interoceptive and attentional systems may contribute to fatigue independently of mood disturbance (Ren et al., 2024).

Altered thalamocortical connectivity has also been shown in fatigue associated with other neurological disorders (de la Cruz et al., 2017; Leitner et al., 2024). In fatigued patients with multiple sclerosis, reduced functional connectivity was identified between the thalamus and both the DLPFC and caudate (de la Cruz et al., 2017). In post-covid fatigue, there was reduced FC between the thalamus and motor areas (Leitner et al., 2024). Further, stroke lesion mapping studies have demonstrated the thalamus as contributing to risk of PSF (Wang et al., 2022). Together, these studies suggest a central role of thalamocortical network disfunction in fatigue pathophysiology.

### 4.3 Broader neural disruption in stroke: amplitude and node strength

As an exploratory analysis, we also examined regional signal amplitude and functional connectivity node strength to characterize broader patterns of neural disruption in stroke participants relative to controls (Figs. 3, S1; Supplementary Tables S4, S5). Although our regional amplitude and node-strength findings did not survive FDR correction and should be interpreted as hypothesis generating, the overall pattern is broadly consistent with prior neuroimaging work suggesting that PSF arises within distributed large-scale network dysfunction rather than a single focal lesion effect. A recent systematic review noted that only a small number of resting-state fMRI studies have examined PSF, but across studies the implicated regions repeatedly include prefrontal, thalamic, insular, and default mode systems (Cotter et al., 2022; Jolly et al., 2023).

In particular, Cotter et al. reported posterior hypoactivity together with medial prefrontal hyperactivity in PSF, interpreting these findings as evidence of dysfunction within fronto-striatal-thalamic and frontal-occipital systems (Cotter et al., 2022). More recent resting-state connectivity work has likewise implicated insular and default mode circuitry in PSF, further supporting involvement of salience and internally directed processing networks (Ren et al., 2024).

Compared to this prior research, the overlap in our sample between elevated fALFF and reduced node strength in the left rostral anterior cingulate is biologically plausible. This region is a key hub of interoceptive processing, and the combination of relatively increased local spontaneous activity with weaker large-scale coupling may reflect compensatory effects (Craig, 2009; Schaechter et al., 2023).

The elevated fALFF observed in VentralDC is also important given evidence linking thalamic and diencephalic systems to fatigue in other neurologic disorders. In MS, fatigue has been associated with abnormal resting-state connectivity of thalamic subregions, and in PSF, newer work has similarly suggested altered thalamocortical organization (de la Cruz et al., 2017; Wang et al., 2024). Together with our ROI-based findings of thalamus-insula and thalamus-ventral attention hyperconnectivity, these exploratory regional results support a model in which PSF reflects broader disruption of salience, arousal, and control-network function (Ren et al., 2024; Wang et al., 2024).

### 4.4 Clinical and translational implications

These findings have several implications for fatigue research and clinical practice. First, the dissociation between fatigue and depression observed in our residualized sensitivity analysis is consistent with fatigue being, to some degree, neurobiologically separable from depression. However, this association did not reach significance in our primary depression-adjusted model and was sensitive to pre-processing. PSF is frequently attributed to depressive symptomatology in clinical practice (Mutai et al., 2017; Wu et al., 2015a). Our data suggest this may not be appropriate for a proportion of patients and that fatigue-specific neural targets should be considered in treatment development.

Second, both frontoparietal and thalamic circuits identified here may represent candidate targets for intervention. Neuromodulation with TMS and tDCS have shown preliminary efficacy for fatigue in other neurological conditions (Chalah et al., 2017; De Doncker et al., 2021; Dong et al., 2021; Ferrucci et al., 2014; Gaede et al., 2018; Mortezanejad et al., 2020; Soltsov et al., 2025), and our findings may eventually provide neuroimaging-based rationale for targeting frontoparietal and thalamocortical circuits in PSF specifically, though associations were sensitive to preprocessing choices in our own analyses and should be replicated before informing intervention design. Future studies combining neuroimaging with brain stimulation in PSF populations are warranted to further investigate these mechanisms. In addition, although direct evidence in PSF is limited, exercise has been shown to modify thalamic and large-scale functional connectivity in other populations, supporting a plausible mechanism where aerobic rehabilitation may improve fatigue through enhanced thalamocortical network efficiency (Sandroff et al., 2025).

Third, our findings raise the hypothesis that PSF may be neurally heterogeneous. Some patients may show primarily affective-motivational circuit involvement (i.e., FP-LIM disruption), while others may show predominantly arousal-interoceptive upregulation (i.e., thalamic hyperconnectivity). This heterogeneity, if confirmed, may partly explain the inconsistent results observed in PSF intervention trials to date (Jolly et al., 2023; Soltsov et al., 2025). Neuroimaging-informed patient stratification may be an important consideration in future trial design to improve treatment response rates and identify responder subgroups.

### 4.5 Limitations

Several limitations should be noted. The SF-36 Vitality subscale is a broad measure that does not distinguish between physical, mental, and cognitive fatigue dimensions (Mead et al., 2007); future work using multidimensional fatigue measures (i.e., separating somatic, cognitive, and motivational components) may allow for finer characterization of neural correlates (Billones et al., 2021; Malloy et al., 2021; Soltsov et al., 2025). The specific mode of SF-36 administration and any accommodations for participants with language, attention, or memory deficits were not documented in the available study records. Therefore, the potential influence of cognitive or communication impairments on SF-36 Vitality scores cannot be fully assessed. In addition, the SF-36 Vitality subscale and our depression measure (GDS-15) share overlapping energy/anergia items; residualizing vitality against depressive symptoms therefore removes some shared variance but cannot fully separate the two constructs, which should be kept in mind when interpreting the findings above. Although the thalamic residualized associations remained significant after removing overlapping energy/anergia content from the depression measure (item level overlap), residualization cannot eliminate broader conceptual or neurobiological overlap between fatigue and depression at the neural level.

The cross-sectional nature of the connectivity-behavior associations within this longitudinal sample are correlational. It is not known whether thalamic hyperconnectivity precedes fatigue development or arises as a consequence. The primary fatigue-connectivity associations were not significant within stroke participants alone after multiple comparisons correction, limiting conclusions about stroke-specific neural mechanisms of fatigue. Further, fatigue may partly reflect pre-existing, stroke-independent risk factors (e.g., small vessel disease burden, deconditioning, sedentary behavior) rather than an effect of the stroke itself, and our design cannot distinguish a stroke-caused mechanism from a comorbid, premorbid one. In addition, a GSR-off sensitivity analysis preserved the direction of the thalamic vitality effects but not their statistical significance, indicating these findings are not fully robust to this preprocessing choice.

Lastly, the amplitude and node strength group differences did not survive FDR correction and should be considered exploratory and hypothesis-generating rather than confirmatory. Lesion site subgroups were small (*n* = 19–40), which may have limited power to detect site-specific effects.

Combined with the fatigue-depression item overlap, vitality-group collinearity, and modest lesion-site subgroup sizes noted above, the connectivity findings should be considered preliminary and hypothesis-generating rather than a robust substrate of post-stroke fatigue. The smaller control sample may have further limited our ability to detect between-group differences or group-specific associations. Replication addressing GSR sensitivity is needed before thalamocortical connectivity can be considered a candidate biomarker or neuromodulation target.

## 5 Conclusion

Fatigue was associated with reduced frontoparietal-limbic and increased thalamocortical functional connectivity, specifically within thalamo-insular and thalamo-VAN circuits, across the full sample of stroke subjects and controls. Thalamic associations were most evident in residualized sensitivity analyses but were attenuated after adjustment for depressive symptoms in the primary analysis and showed sensitivity to preprocessing choices. Lesion site and volume did not moderate these associations in the stroke cohort. Together, these findings suggest that fatigue and depressive symptoms may involve overlapping and potentially distinct neural correlates, while providing no evidence that the observed connectivity patterns are stroke-specific. Further investigation of these circuits as markers of fatigue across clinical populations, and their relationship to stroke pathology, is warranted.

## Declaration of generative AI and AI-assisted technologies in the manuscript preparation process

During the preparation of this work the author(s) used ChatGPT and Claude in order to generate and refine code in R for statistical analysis. After using this tool/service, the author(s) reviewed and edited the content as needed and take(s) full responsibility for the content of the published article.

## CRediT authorship contribution statement

**Joan Stilling:** Writing – review & editing, Writing – original draft, Validation, Supervision, Software, Methodology, Funding acquisition, Formal analysis, Conceptualization. **Christie Gillies:** Writing – review & editing, Formal analysis, Data curation. **Ji Hyun Kim:** Writing – review & editing, Visualization. **Keith Jamison:** Writing – review & editing, Formal analysis, Data curation. **Amy Kuceyeski:** Writing – review & editing, Methodology, Funding acquisition, Conceptualization.

## Ethics declaration

Written informed consent to take part in the study and to publish the article has been obtained from all participants or their legal representatives. The privacy rights of participants have been observed.

This study was performed in compliance with relevant laws, regulatory frameworks and guidelines where the research took place. Ethics committee approval was not required under relevant laws and institutional guidelines. Publicly available data - WashU database

## Funding

Supported by AHA - 25CDA1449102 (Joan Stilling) and institutional departmental funds.

## Declaration of competing interest

Joan Stilling - investigator-initiated research support for a clinical trial evaluating noninvasive brain stimulation for post-stroke fatigue, outside the submitted work.

## Data Availability

I have shared a link to the code.
