# Supplementary material for: Thalamocortical connectivity associated with fatigue after accounting for depressive symptoms in stroke survivors and controls
Source: Neuroimage Clin. 2026 Oct 8;52:104063. doi: 10.1016/j.nicl.2026.104063 (PMC13677772; doi:10.1016/j.nicl.2026.104063)
Supplement: Supplementary file 2 [file mmc2.docx]

**Figure S2. Stroke-related differences in regional amplitude.** Surface maps display t-values for the stroke vs. control group contrast from depression-adjusted mixed-effects models (GDS-15 covariate included), shown for (A) ALFF and (B) fALFF. Warm colors (red) indicate greater values in stroke relative to controls; cool colors (blue) indicate reduced values. Color scale reflects t-values. Green outlines denote regions with uncorrected p < 0.05; no regions survived FDR correction. ALFF showed no nominally significant group differences in any region. fALFF was elevated in stroke relative to controls in 10 regions at uncorrected threshold (p < 0.05), including right insula (t = 2.75, p = 0.006), bilateral rostral anterior cingulate cortex (right: t = 2.17, p = 0.030; left: t = 2.64, p = 0.008), bilateral parahippocampal cortex (right: t = 2.17, p = 0.030; left: t = 2.05, p = 0.040), left hippocampus (t = 2.36, p = 0.018), bilateral ventral DC (right: t = 2.29, p = 0.022; left: t = 2.12, p = 0.034), right pars orbitalis (t = 2.15, p = 0.032), and right transverse temporal cortex (t = 2.08, p = 0.038). ALFF = amplitude of low-frequency fluctuations; fALFF = fractional ALFF
