# Supplementary material for: Thalamocortical connectivity associated with fatigue after accounting for depressive symptoms in stroke survivors and controls
Source: Neuroimage Clin. 2026 Oct 8;52:104063. doi: 10.1016/j.nicl.2026.104063 (PMC13677772; doi:10.1016/j.nicl.2026.104063)
Supplement: Supplementary file 3 [file mmc3.docx]

**Suppementary Methods**

The parcels were mapped onto a Yeo-7 partition with the seven canonical cortical Yeo networks plus two additional non-cortical groupings (subcortical structures, cerebellum) needed to assign every fs86 region a network label. The fs86 parcellation itself combines 18 bilateral FreeSurfer aseg subcortical regions with 68 bilateral Desikan-Killiany aparc cortical regions (86 total), each assigned to one of the 9 networks via a fixed, pre-specified lookup table. Within-network connectivity is the unweighted mean of all pairwise edges among the ROIs assigned to that network (upper triangle only, excluding the diagonal); between-network connectivity is the unweighted mean of all pairwise edges connecting the two networks' full ROI sets (not restricted to a triangle, since the two ROI sets differ); and seed-based connectivity (including the thalamus-insula and thalamus-VAN edges) is computed the same way (e.g., thalamus-insula connectivity is the unweighted mean across all bilateral thalamus to bilateral insula edges).

**region_names** = [

"Left-Cerebellum-Cortex", "Left-Thalamus-Proper", "Left-Caudate",

"Left-Putamen", "Left-Pallidum", "Left-Hippocampus", "Left-Amygdala",

"Left-Accumbens-area", "Left-VentralDC", "Right-Cerebellum-Cortex",

"Right-Thalamus-Proper", "Right-Caudate", "Right-Putamen", "Right-Pallidum",

"Right-Hippocampus", "Right-Amygdala", "Right-Accumbens-area", "Right-VentralDC",

"ctx-lh-bankssts", "ctx-lh-caudalanteriorcingulate", "ctx-lh-caudalmiddlefrontal",

"ctx-lh-cuneus", "ctx-lh-entorhinal", "ctx-lh-fusiform", "ctx-lh-inferiorparietal",

"ctx-lh-inferiortemporal", "ctx-lh-isthmuscingulate", "ctx-lh-lateraloccipital",

"ctx-lh-lateralorbitofrontal", "ctx-lh-lingual", "ctx-lh-medialorbitofrontal",

"ctx-lh-middletemporal", "ctx-lh-parahippocampal", "ctx-lh-paracentral",

"ctx-lh-parsopercularis", "ctx-lh-parsorbitalis", "ctx-lh-parstriangularis",

"ctx-lh-pericalcarine", "ctx-lh-postcentral", "ctx-lh-posteriorcingulate",

"ctx-lh-precentral", "ctx-lh-precuneus", "ctx-lh-rostralanteriorcingulate",

"ctx-lh-rostralmiddlefrontal", "ctx-lh-superiorfrontal", "ctx-lh-superiorparietal",

"ctx-lh-superiortemporal", "ctx-lh-supramarginal", "ctx-lh-frontalpole",

"ctx-lh-temporalpole", "ctx-lh-transversetemporal", "ctx-lh-insula",

"ctx-rh-bankssts", "ctx-rh-caudalanteriorcingulate", "ctx-rh-caudalmiddlefrontal",

"ctx-rh-cuneus", "ctx-rh-entorhinal", "ctx-rh-fusiform", "ctx-rh-inferiorparietal",

"ctx-rh-inferiortemporal", "ctx-rh-isthmuscingulate", "ctx-rh-lateraloccipital",

"ctx-rh-lateralorbitofrontal", "ctx-rh-lingual", "ctx-rh-medialorbitofrontal",

"ctx-rh-middletemporal", "ctx-rh-parahippocampal", "ctx-rh-paracentral",

"ctx-rh-parsopercularis", "ctx-rh-parsorbitalis", "ctx-rh-parstriangularis",

"ctx-rh-pericalcarine", "ctx-rh-postcentral", "ctx-rh-posteriorcingulate",

"ctx-rh-precentral", "ctx-rh-precuneus", "ctx-rh-rostralanteriorcingulate",

"ctx-rh-rostralmiddlefrontal", "ctx-rh-superiorfrontal", "ctx-rh-superiorparietal",

"ctx-rh-superiortemporal", "ctx-rh-supramarginal", "ctx-rh-frontalpole",

"ctx-rh-temporalpole", "ctx-rh-transversetemporal", "ctx-rh-insula"

]

**network_labels** = [9,8,8,8,8,8,8,8,8,9,8,8,8,8,8,8,8,8,

7,4,7,1,5,1,7,5,7,1,5,1,5,7,1,2,7,7,7,

1,2,4,2,7,7,6,7,3,2,4,5,5,2,4,2,4,6,1,5,

1,7,5,7,1,5,1,5,7,1,2,6,7,6,1,2,4,2,7,7,

6,7,3,2,4,5,5,2,4]

**network_mapping** = {

1: "VIS",

2: "SOM",

3: "DAN",

4: "VAN",

5: "LIM",

6: "FP",

7: "DMN",

8: "SUB",

9: "CER"

}
