# Supplementary material for: Thalamocortical connectivity associated with fatigue after accounting for depressive symptoms in stroke survivors and controls
Source: Neuroimage Clin. 2026 Oct 8;52:104063. doi: 10.1016/j.nicl.2026.104063 (PMC13677772; doi:10.1016/j.nicl.2026.104063)
Supplement: Supplementary file 4 [file mmc4.docx]

**SUPPLEMENTARY TABLES**

**Table S1. Vitality-imaging nominal associations: depression-adjusted primary model (n = 201 observations, 132 subjects)**

| **Model family** | **Feature** | **Term** | **β** | **SE** | **t** | **p** | **FDR q** |  | **n obs** | **n subj** |
| --- | --- | --- | --- | --- | --- | --- | --- | --- | --- | --- |
| **Between-network functional connectivity** | | | | | | | | | | |
|  | LIM-FP | Vitality | 0.0013 | 0.0004 | 3.345 | 0.001 | 0.048 | ***** | 201 | 132 |
|  | LIM-FP | Vitality x Group (stroke) | -0.0012 | 0.0004 | -3.110 | 0.002 | 0.103 | + | 201 | 132 |
|  | DAN-SUB | Vitality x Group (stroke) | -0.0016 | 0.0006 | -2.493 | 0.014 | 0.262 | + | 201 | 132 |
|  | VIS-DMN | Vitality x Group (stroke) | 0.0010 | 0.0004 | 2.390 | 0.018 | 0.262 | + | 201 | 132 |
|  | LIM-SUB | Vitality | -0.0007 | 0.0003 | -2.318 | 0.022 | 0.371 | + | 201 | 132 |
|  | DAN-SUB | Vitality | 0.0014 | 0.0006 | 2.278 | 0.024 | 0.371 | + | 201 | 132 |
|  | VIS-DMN | Vitality | -0.0009 | 0.0004 | -2.105 | 0.037 | 0.380 | + | 201 | 132 |
|  | SOM-LIM | Vitality | 0.0006 | 0.0003 | 1.981 | 0.050 | 0.380 | + | 201 | 132 |
|  | within SUB | Vitality x Group (stroke) | 0.0011 | 0.0005 | 2.299 | 0.023 | 0.262 | + | 201 | 132 |
|  | within SUB | Vitality | -0.0010 | 0.0005 | -2.053 | 0.042 | 0.380 | + | 201 | 132 |
| **Thalamic ROI-based connectivity** | | | | | | | | | | |
|  | Thalamus → Insula | Vitality | -0.0042 | 0.0014 | -2.987 | 0.003 | 0.075 | + | 201 | 132 |
|  | Thalamus → Caudal ACC | Vitality | -0.0031 | 0.0013 | -2.331 | 0.021 | 0.241 | + | 201 | 132 |
|  | Thalamus → Caudal ACC | Vitality x Group (stroke) | 0.0028 | 0.0014 | 2.101 | 0.037 | 0.635 | + | 201 | 132 |
|  | Thalamus → Insula | Vitality x Group (stroke) | 0.0029 | 0.0014 | 2.058 | 0.041 | 0.635 | + | 201 | 132 |
|  | Thalamus → VAN | Vitality | -0.0029 | 0.0009 | -3.128 | 0.002 | 0.075 | + | 201 | 132 |
|  | Insula → SUB | Vitality | -0.0016 | 0.0006 | -2.439 | 0.016 | 0.241 | + | 201 | 132 |
|  | Thalamus → VAN | Vitality x Group (stroke) | 0.0022 | 0.0009 | 2.401 | 0.018 | 0.635 | + | 201 | 132 |
| **DLPFC ROI-based connectivity** | | | | | | | | | | |
|  | DLPFC → LIM | Vitality x Group (stroke) | -0.0015 | 0.0005 | -2.696 | 0.008 | 0.378 | + | 201 | 132 |
|  | DLPFC → LIM | Vitality | 0.0013 | 0.0005 | 2.380 | 0.019 | 0.575 | + | 201 | 132 |
|  | DLPFC → LH frontalpole | Vitality | 0.0045 | 0.0015 | 3.043 | 0.003 | 0.258 | + | 201 | 132 |
|  | DLPFC → RH frontalpole | Vitality x Group (stroke) | -0.0046 | 0.0017 | -2.646 | 0.009 | 0.378 | + | 201 | 132 |
|  | DLPFC → RH frontalpole | Vitality | 0.0044 | 0.0017 | 2.562 | 0.011 | 0.529 | + | 201 | 132 |
|  | DLPFC → LH frontalpole | Vitality x Group (stroke) | -0.0037 | 0.0015 | -2.540 | 0.012 | 0.378 | + | 201 | 132 |
|  | DLPFC → RH isthmuscingulate | Vitality | 0.0027 | 0.0012 | 2.220 | 0.028 | 0.648 | + | 201 | 132 |
|  | DLPFC → RH inferiortemporal | Vitality x Group (stroke) | -0.0028 | 0.0013 | -2.199 | 0.029 | 0.686 | + | 201 | 132 |
|  | DLPFC → RH inferiorparietal | Vitality x Group (stroke) | -0.0028 | 0.0014 | -2.032 | 0.044 | 0.774 | + | 201 | 132 |
|  | DLPFC → LH isthmuscingulate | Vitality | 0.0026 | 0.0013 | 1.978 | 0.050 | 0.925 | + | 201 | 132 |
|  |  |  |  |  |  |  |  |  | |  |
| *Models: Imaging Feature(ij) = b0 + b1*Vitality(ij) + b2*GDS-15(ij) + b3*Group(i) + b4*(Vitality x Group)(ij) + b5*Age(i) + b6*Sex(i) + b7*Time(ij) + u(i) + e(ij). FDR correction applied within model family (Benjamini-Hochberg). * FDR q < 0.05; + uncorrected p < 0.05 only. Vitality scored such that lower values = greater fatigue. GDS-15 = Geriatric Depression Scale Score.* | | | | | | | | | | |

**Table S2. Vitality-imaging nominal associations: residualized vitality sensitivity analysis (n = 201 observations, 132 subjects)**

| **Model family** | **Feature** | **Term** | **β** | **SE** | **t** | **p** | **FDR q** |  | **n obs** | **n subj** |
| --- | --- | --- | --- | --- | --- | --- | --- | --- | --- | --- |
| **Between-network functional connectivity** | | | | | | | | | | |
|  | LIM-SUB | Vitality (residualized) | -0.0011 | 0.0004 | -2.636 | 0.009 | 0.389 | + | 201 | 132 |
|  | LIM-DMN | Vitality (residualized) | 0.0008 | 0.0003 | 2.382 | 0.018 | 0.389 | + | 201 | 132 |
|  | LIM-SUB | Vitality (residualized) x Group (stroke) | 0.0010 | 0.0004 | 2.311 | 0.022 | 0.732 | + | 201 | 132 |
|  | VIS-DMN | Vitality (residualized) x Group (stroke) | 0.0013 | 0.0006 | 2.157 | 0.032 | 0.732 | + | 201 | 132 |
|  | LIM-FP | Vitality (residualized) | 0.0011 | 0.0005 | 2.143 | 0.033 | 0.389 | + | 201 | 132 |
|  | VAN-SUB | Vitality (residualized) | -0.0012 | 0.0006 | -2.137 | 0.034 | 0.389 | + | 201 | 132 |
|  | VIS-DMN | Vitality (residualized) | -0.0011 | 0.0006 | -2.030 | 0.044 | 0.402 | + | 201 | 132 |
|  | LIM-DMN | Vitality (residualized) x Group (stroke) | -0.0007 | 0.0004 | -1.982 | 0.049 | 0.732 | + | 201 | 132 |
| **Thalamic ROI-based connectivity** | | | | | | | | | | |
|  | Thalamus → Insula | Vitality (residualized) | -0.0071 | 0.0019 | -3.751 | <0.001 | 0.011 | ***** | 201 | 132 |
|  | Thalamus → Insula | Vitality (residualized) x Group (stroke) | 0.0062 | 0.0020 | 3.066 | 0.002 | 0.083 | + | 201 | 132 |
|  | Thalamus → VAN | Vitality (residualized) | -0.0043 | 0.0012 | -3.518 | <0.001 | 0.012 | ***** | 201 | 132 |
|  | Thalamus → VAN | Vitality (residualized) x Group (stroke) | 0.0039 | 0.0013 | 2.945 | 0.004 | 0.083 | + | 201 | 132 |
|  | Insula → SUB | Vitality (residualized) | -0.0023 | 0.0009 | -2.731 | 0.007 | 0.106 | + | 201 | 132 |
|  | Thalamus → SOM | Vitality (residualized) | -0.0034 | 0.0017 | -2.052 | 0.042 | 0.462 | + | 201 | 132 |
| **DLPFC ROI-based connectivity** | | | | | | | | | | |
|  | DLPFC → LIM | Vitality (residualized) x Group (stroke) | -0.0016 | 0.0008 | -2.075 | 0.039 | 0.969 | + | 201 | 132 |
|  | DLPFC → RH inferiortemporal | Vitality (residualized) x Group (stroke) | -0.0043 | 0.0018 | -2.375 | 0.019 | 0.969 | + | 201 | 132 |
|  | DLPFC → LH frontalpole | Vitality (residualized) | 0.0043 | 0.0020 | 2.162 | 0.032 | 0.990 | + | 201 | 132 |
|  | DLPFC → RH frontalpole | Vitality (residualized) | 0.0050 | 0.0023 | 2.151 | 0.033 | 0.990 | + | 201 | 132 |
|  | DLPFC → RH inferiortemporal | Vitality (residualized) | 0.0036 | 0.0017 | 2.110 | 0.036 | 0.990 | + | 201 | 132 |
|  | DLPFC → RH frontalpole | Vitality (residualized) x Group (stroke) | -0.0052 | 0.0025 | -2.091 | 0.038 | 0.969 | + | 201 | 132 |
|  | DLPFC → RH middletemporal | Vitality (residualized) x Group (stroke) | -0.0036 | 0.0017 | -2.053 | 0.042 | 0.969 | + | 201 | 132 |
|  |  |  |  |  |  |  |  |  | |  |
| *Vitality residualized with respect to GDS-15 and covariates prior to modeling: Vitality(resid) = residuals(Vitality ~ GDS-15 + Age + Sex + Group + Time). Models: Imaging Feature(ij) = b0 + b1*Vitality_resid(ij) + b2*GDS-15(ij) + b3*Group(i) + b4*(Vitality_resid x Group)(ij) + b5*Age(i) + b6*Sex(i) + b7*Time(ij) + u(i) + e(ij). FDR correction applied within model family (Benjamini-Hochberg). * FDR q < 0.05; + uncorrected p < 0.05 only.* | | | | | | | | | | |

**Table S3. Structural disconnection moderation nominal associations: Vitality x ChaCo interaction (stroke participants only)**

| **Model family** | **Feature** | **Term** | **β** | **SE** | **t** | **p** | **FDR q** |  | **n obs** | **n subj** |
| --- | --- | --- | --- | --- | --- | --- | --- | --- | --- | --- |
| **Between-network functional connectivity** | | | | | | | | | | |
|  | VAN-SUB | Vitality x ChaCo (interaction) | 0.0133 | 0.0051 | 2.609 | 0.010 | 0.330 | + | 128 | 86 |
|  | LIM-FP | Vitality x ChaCo (interaction) | 0.0107 | 0.0043 | 2.490 | 0.014 | 0.330 | + | 128 | 86 |
| **Thalamic ROI-based connectivity** | | | | | | | | | | |
|  | Insula → DAN | Vitality x ChaCo (interaction) | -0.0326 | 0.0157 | -2.074 | 0.041 | 0.657 | + | 128 | 86 |

*Stroke-only moderation models restricted to between-network FC and ROI-based FC families. Vitality, ChaCo, and GDS-15 z-scored within stroke sample. Model: Imaging Feature(ij) = b0 + b1*behav_z(ij) + b2*chaco_z(i) + b3*GDS-15_z(ij) + b4*(behav_z x chaco_z)(ij) + b5*Age(i) + b6*Sex(i) + b7*Time(ij) + u(i) + e(ij). Only the behav_z x chaco_z interaction term is shown. FDR correction applied within model family (Benjamini-Hochberg). * FDR q < 0.05; + uncorrected p < 0.05 only.*

| **Table S4. FC node strength group differences: stroke vs. control (GDS-15-adjusted, uncorrected p < 0.05)** | | | | | | |  |  |  |  |
| --- | --- | --- | --- | --- | --- | --- | --- | --- | --- | --- |
| **Metric** | **Region** | **t** | **p** | **FDR q** |  | **Est.** | **SE** | **n obs** | **n subj** |  |
| **FC Node Strength** | | | | | | | | | | |
|  | LH insula | -2.683 | 0.007 | 0.593 | + | -0.0164 | 0.0061 | 201 | 132 |  |
|  | LH rostralanteriorcingulate | -2.314 | 0.021 | 0.593 | + | -0.0146 | 0.0063 | 201 | 132 |  |
|  | Left-Accumbens-area | -2.309 | 0.021 | 0.593 | + | -0.0122 | 0.0053 | 201 | 132 |  |
|  | RH superiorfrontal | -2.168 | 0.030 | 0.593 | + | -0.0156 | 0.0072 | 201 | 132 |  |
|  | RH medialorbitofrontal | -2.115 | 0.034 | 0.593 | + | -0.0143 | 0.0068 | 201 | 132 |  |
|  |  |  |  |  |  |  |  |  |  |  |
|  | | | | | | | | | |  |

*Group differences in parcel-wise FC node strength (sum of Fisher-z transformed correlations) adjusted for depressive symptoms (GDS-15), age, sex, and time. Positive t-values indicate greater strength in stroke relative to controls. No regions survived FDR correction. + uncorrected p < 0.05 only.*

| **Table S5. ALFF and fALFF group differences: stroke vs. control (GDS-15-adjusted, uncorrected p < 0.05)** | | | | | | |  |  |  |  |
| --- | --- | --- | --- | --- | --- | --- | --- | --- | --- | --- |
| **Metric** | **Region** | **t** | **p** | **FDR q** |  | **Est.** | **SE** | **n obs** | **n subj** |  |
| **ALFF** No nominally significant regions | | | | | | | | | | |
| **fALFF** | | | | | | | | | | |
|  | fALFF RH insula | 2.747 | 0.006 | 0.283 | + | 0.0187 | 0.0068 | 169 | 114 |  |
|  | fALFF LH rostralanteriorcingulate | 2.639 | 0.008 | 0.283 | + | 0.0177 | 0.0067 | 169 | 114 |  |
|  | fALFF Left-Hippocampus | 2.357 | 0.018 | 0.283 | + | 0.0127 | 0.0054 | 169 | 114 |  |
|  | fALFF Right-VentralDC | 2.294 | 0.022 | 0.283 | + | 0.0161 | 0.0070 | 169 | 114 |  |
|  | fALFF RH rostralanteriorcingulate | 2.174 | 0.030 | 0.283 | + | 0.0161 | 0.0074 | 169 | 114 |  |
|  | fALFF RH parahippocampal | 2.169 | 0.030 | 0.283 | + | 0.0147 | 0.0068 | 169 | 114 |  |
|  | fALFF RH parsorbitalis | 2.145 | 0.032 | 0.283 | + | 0.0127 | 0.0059 | 169 | 114 |  |
|  | fALFF Left-VentralDC | 2.124 | 0.034 | 0.283 | + | 0.0143 | 0.0067 | 169 | 114 |  |
|  | fALFF RH transversetemporal | 2.076 | 0.038 | 0.283 | + | 0.0167 | 0.0081 | 169 | 114 |  |
|  | fALFF LH parahippocampal | 2.051 | 0.040 | 0.283 | + | 0.0136 | 0.0066 | 169 | 114 |  |
|  |  |  |  |  |  |  |  |  |  |  |

**Table S6. Sensitivity analyses for key thalamic connectivity findings (item-overlap exclusion, head-motion covariate, and GSR-off preprocessing; primary GSR-on, residualized-vitality model unless noted)**

| **Model family** | **Feature** | **Term** | **β** | **SE** | **t** | **p** | **FDR q** |  | **n obs** | **n subj** |
| --- | --- | --- | --- | --- | --- | --- | --- | --- | --- | --- |
| **Item-overlap sensitivity: GDS-15 item 13 ("Do you feel full of energy?") excluded, vitality residualized against reduced depression score** | | | | | | | | | | |
|  | Thalamus-Insula | Vitality (original, full-item) | -0.0071 | 0.0019 | -3.75 | 0.0002 | 0.0110 | * | 201 | 132 |
|  | Thalamus-Insula | Vitality (item-13-excluded) | -0.0066 | 0.0018 | -3.63 | 0.0004 | 0.0156 | * | 201 | 132 |
|  | Thalamus-VAN | Vitality (original, full-item) | -0.0043 | 0.0012 | -3.51 | 0.0001 | 0.0124 | * | 201 | 132 |
|  | Thalamus-VAN | Vitality (item-13-excluded) | -0.0041 | 0.0012 | -3.45 | 0.0001 | 0.0156 | * | 201 | 132 |
| **Head-motion sensitivity: subject-level mean framewise displacement (FD) added as covariate to primary (uncentered, GSR-on) model** | | | | | | | | | | |
|  | Thalamus-Insula | Vitality (without FD) | -0.0042 | 0.0015 | -2.90 | 0.0044 | 0.0087 | * | 201 | 132 |
|  | Thalamus-Insula | Vitality (with FD covariate) | -0.0044 | 0.0018 | -2.54 | 0.0126 | 0.0252 | * | 169 | 114 |
|  | Thalamus-Insula | Vitality × Group (without FD) | 0.0029 | 0.0015 | 1.99 | 0.0485 | 0.0485 | * | 201 | 132 |
|  | Thalamus-Insula | Vitality × Group (with FD covariate) | 0.0031 | 0.0018 | 1.73 | 0.0870 | 0.0870 | + | 169 | 114 |
|  | Thalamus-VAN | Vitality (without FD) | -0.0029 | 0.0009 | -3.05 | 0.0027 | 0.0087 | * | 201 | 132 |
|  | Thalamus-VAN | Vitality (with FD covariate) | -0.0031 | 0.0011 | -2.75 | 0.0069 | 0.0252 | * | 169 | 114 |
|  | Thalamus-VAN | Vitality × Group (without FD) | 0.0022 | 0.0010 | 2.36 | 0.0198 | 0.0264 | * | 201 | 132 |
|  | Thalamus-VAN | Vitality × Group (with FD covariate) | 0.0026 | 0.0011 | 2.27 | 0.0250 | 0.0333 | * | 169 | 114 |
|  | - | FDmean itself (thalamus-insula model) | 0.108 | 0.182 | 0.591 | 0.5560 | - |  | 169 | 114 |
|  | - | FDmean itself (thalamus-VAN model) | 0.0749 | 0.114 | 0.658 | 0.5120 | - |  | 169 | 114 |
| **GSR sensitivity: identical subjects/observations refit on GSR-off connectivity estimates (pre-registered GSR-on = primary pipeline)** | | | | | | | | | | |
|  | Thalamus-Insula | Vitality (GSR-on) | -0.0042 | 0.0015 | -2.90 | 0.0044 | 0.0087 | * | 201 | 132 |
|  | Thalamus-Insula | Vitality (GSR-off) | -0.0033 | 0.0022 | -1.50 | 0.1350 | 0.2710 |  | 201 | 132 |
|  | Thalamus-Insula | Vitality × Group (GSR-on) | 0.0029 | 0.0015 | 1.99 | 0.0485 | 0.0485 | * | 201 | 132 |
|  | Thalamus-Insula | Vitality × Group (GSR-off) | 0.0011 | 0.0022 | 0.493 | 0.6230 | 0.6230 |  | 201 | 132 |
|  | Thalamus-VAN | Vitality (GSR-on) | -0.0029 | 0.0009 | -3.05 | 0.0027 | 0.0087 | * | 201 | 132 |
|  | Thalamus-VAN | Vitality (GSR-off) | -0.0031 | 0.0020 | -1.54 | 0.1270 | 0.2710 |  | 201 | 132 |
|  | Thalamus-VAN | Vitality × Group (GSR-on) | 0.0022 | 0.0010 | 2.36 | 0.0198 | 0.0264 | * | 201 | 132 |
|  | Thalamus-VAN | Vitality × Group (GSR-off) | 0.0013 | 0.0021 | 0.606 | 0.5450 | 0.6230 |  | 201 | 132 |

** = survives FDR correction (q < .05); + = nominally significant (p < .05) but not surviving FDR correction.*

**Table S7. Collinearity diagnostics (variance inflation factors) for the primary model predictor set (functional-connectivity family, applies to both thalamus-insula and thalamus-VAN models)**

| **Term** | **VIF (uncentered)** | **VIF (vitality mean-centered)** | **Change** |
| --- | --- | --- | --- |
| **Vitality (behav)** | 6.52 | 6.52 | unchanged |
| **Group (stroke)** | 8.83 | 1.50 | reduced ~6-fold |
| **Vitality × Group (interaction)** | 10.39 | 4.90 | reduced ~2-fold, now below conventional VIF=5 threshold |
| **Depression score (GDS-15_score)** | 1.90 | 1.90 | unchanged (unremarkable throughout) |
| **Age** | 1.01 | 1.01 | unchanged (unremarkable throughout) |
| **Sex** | 1.12 | 1.12 | unchanged (unremarkable throughout) |
| **Time (time_mo)** | 1.31 | 1.31 | unchanged (unremarkable throughout) |

*VIF > 5 indicates meaningful collinearity; VIF > 10 is conventionally treated as severe. The elevated VIF for group and the vitality-by-group interaction reflects that mean vitality differs systematically between stroke and control participants in this sample (a substantive feature of the data, not a specification artifact); mean-centering vitality before forming the interaction term reduces but does not eliminate this. Depression score's own VIF (1.90) confirms the vitality-depression correlation is not the source of the collinearity. Values shown are from the functional-connectivity model family (which includes both thalamic edges in this table)*
